# Loss of *BAP1* as a candidate predictive biomarker for immunotherapy of mesothelioma

**DOI:** 10.1186/s13073-019-0631-0

**Published:** 2019-03-26

**Authors:** Marc Ladanyi, Francisco Sanchez Vega, Marjorie Zauderer

**Affiliations:** 1 Department of Pathology and Human Oncology and Pathogenesis Program, Memorial Sloan Kettering Cancer Center, 1275 York Avenue, New York, NY 10065 USA; 2 Computational Biology Program, Memorial Sloan Kettering Cancer Center, 1275 York Avenue, New York, NY 10065 USA; 3 Department of Medicine, Memorial Sloan Kettering Cancer Center, 1275 York Avenue, New York, NY 10065 USA

## Abstract

As trials of immune checkpoint inhibitor (ICI) therapies demonstrate responses in only a minority of pleural mesotheliomas (PlMs) and largely exclude patients with the related peritoneal mesothelioma (PeM), clinicians need predictive biomarkers of response and inclusion of PeM patients in future trials. A new study finds that loss of the deubiquitinase BAP1 in PeM correlates with an inflammatory tumor microenvironment, suggesting that *BAP1* status might identify PeM, and possibly PlM, patients who would benefit from ICI therapy.

## Mesothelioma—a rare and challenging cancer

Malignant peritoneal mesothelioma (PeM), an aggressive cancer arising from the mesothelial lining of the abdominal cavity, is at least ten times less common than its counterpart in the chest cavity—malignant pleural mesothelioma (PlM)—and the proportion of cases etiologically attributable to exposure to asbestos appears lower than for the latter. While the initial 2011 report of frequent somatic inactivation in PlM of the gene encoding the ubiquitin carboxyl-terminal hydrolase BAP1 [1] was followed by the finding that PeM shows a similar high prevalence of *BAP1* alterations [2], PeM also shows some genetic differences, notably a lower prevalence of losses of other tumor suppressors—*CDKN2A* and *NF2*—than PlM. Somatic *BAP1* mutations are also seen not infrequently in carcinomas of the kidney and intrahepatic bile ducts and in ocular melanomas. BAP1 is a nuclear deubiquitinase that regulates the ubiquitination of select histones, transcription factors, and other nuclear proteins. Only modest survival improvements are obtained with standard treatments for both PlM and PeM. For early and locally advanced disease, aggressive multi-modality therapy is pursued, including surgery and cytotoxic chemotherapy. This has resulted in a median overall survival approaching 3 years for pleural disease and 5 years for peritoneal disease. Advanced disease is not amenable to macroscopic complete resection and is treated with systemic therapy, which improves median overall survival by approximately 3 months, from 9 to 16 months to 12 to 18 months.

Given these disappointing statistics, there has been intense interest in evaluating new immunotherapy approaches for this tumor type. In a recent study published in *Genome Medicine*, Shrestha and colleagues [3] perform an integrated genomic, transcriptomic, and proteomic analysis of 19 PeM cases. Specifically, they performed gene-set-enrichment analysis of mRNA and protein expression data, comparing *BAP1*-altered versus *BAP1*-intact tumors; this identified, among other differences between these two groups, a striking difference in immune-system-associated pathways, with *BAP1*-altered tumors showing signatures of cytokine signaling and of the innate immune system. The investigators went on to show that *BAP1* loss in PeM is associated with a more inflamed tumor microenvironment and propose that this finding could be useful as a predictive marker of responsiveness to immune checkpoint inhibitors (ICIs).

## Immunotherapy trials and tribulations for mesothelioma

To date, clinical data on ICIs in PeM remain quite sparse. While some trials have demonstrated efficacy of anti-PD-1 and anti-PD-L1 therapy in mesothelioma, which target programmed cell death protein 1 and programmed cell death 1 ligand 1, respectively, the representation of PeM cases in these studies has been very limited owing to their relative rarity. In the large negative randomized DETERMINE trial of tremelimumab [antibody against cytotoxic T-lymphocyte associated protein 4 (CTLA-4)] versus placebo [4], PeM was included, but only 18 patients with peritoneal disease were enrolled (out of 571 total mesothelioma patients), thereby preventing subgroup analysis for efficacy specifically in PeM. The lack of efficacy observed in the DETERMINE trial might have been due to the single-agent use of anti-CTLA-4 as well as the specific anti-CTLA-4 agent selected. More recent mesothelioma immunotherapy trials such as KEYNOTE-028 (anti-PD-1) and IFCT-1501 MAPS2 (anti-PD-1 monotherapy or combined with anti-CTLA-4) have excluded patients with a peritoneal primary site. Notably, in these trials, the agents used—pembrolizumab and nivolumab with and without ipilimumab, respectively—have demonstrated response rates ranging from 20 to 31%. Furthermore, PlM immunotherapy trials have not simultaneously developed or reported on predictive biomarkers that might facilitate improved patient selection.

PD-L1 expression levels as well as high tumor mutational burden (TMB) have been intensely investigated and shown some utility as predictors of ICI responses in different cancers [5]. In PlM, a trend associating high PD-L1 expression and a higher response rate has been reported, warranting further investigation. Given the modest activity in PlM of the currently available checkpoint inhibitors, predictive markers beyond PD-L1 and TMB are necessary to identify patients most likely to derive benefit from checkpoint inhibition, a need made even more pressing by the fact that TMB is notably low in PlM [6], as is also the case for PeM, as shown in the present study**.**

**Fig. 1:**
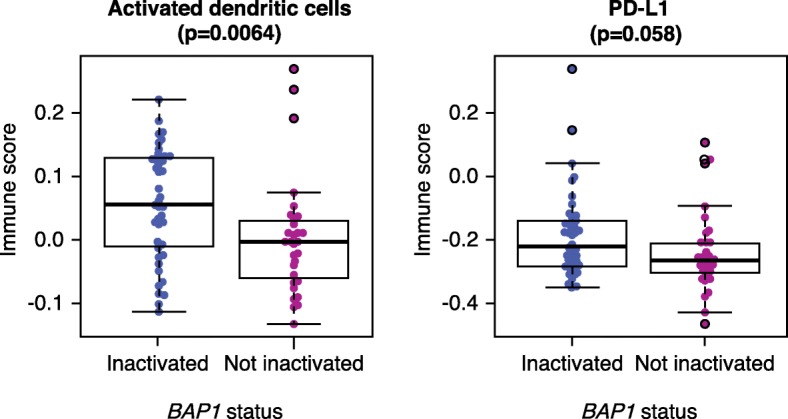
Comparison of immune infiltration scores for activated dendritic cells (*left*) and PD-L1 expression levels (*right*) as a function of *BAP1* inactivation status in 74 PlM samples from the TCGA cohort [6]. *BAP1* status was assessed as described by Hmeljak et al. [6]. The immune scores were computed using the single-sample gene-set-enrichment analysis (ssGSEA) and the immune infiltrate gene signatures from Bindea et al. [10]. Reported *p* values are based on a two-sided Wilcoxon rank-sum test. *BAP1* ubiquitin carboxyl-terminal hydrolase BAP1, *PD-L1* programmed cell death 1 ligand 1, *PlM* pleural mesothelioma

In other tumors, such as non-small cell lung cancer, immunotherapy given in combination with cytotoxic chemotherapy is emerging as the preferred treatment approach for tumors that are TMB low and PD-L1 low or negative. Evaluation of this combination approach in mesothelioma is ongoing in the PreCOG trial (NCT0289919). Based on the data from Shrestha and colleagues [3], examination of *BAP1* status in relation to immunotherapy response in mesothelioma is warranted. Interestingly, in uveal melanoma, another disease with frequent *BAP1* loss, the loss of *BAP1* expression is associated with an increased infiltration of CD3^+^ and CD8^+^ T cells [7], a finding paralleled in PeM tissues by the investigations of Shrestha and colleagues [3]. Whether *BAP1* loss might be more broadly applicable across different cancer types as a biomarker for an immune-inflamed tumor microenvironment will require further studies. Shrestha and colleagues also report higher expression of several immune checkpoint molecules, including PD-L1 (*CD274*) in *BAP1*-altered PeM. In light of this, we re-analyzed the TCGA PlM data according to *BAP1* status and found a similar but sub-significant trend for PD-L1 (*CD274*) to be higher in *BAP1*-altered samples, but the most significant association was for the mRNA signature of activated dendritic cells to be more prominent in the *BAP1*-altered group (Fig. 1).

## Remaining challenges and future prospects

While the potential link between *BAP1* loss—a known driver of mesothelioma—with response to ICI treatments is intriguing, an important caveat is that *BAP1* resides at 3p21.1, a chromosomal region that also contains two other genes encoding epigenetic regulators, *PBRM1* and *SETD2*. In this respect, the proximity of *BAP1* and *PBRM1* and their frequent co-inactivation (or co-haploinsufficiency) in PeM might represent a confounding factor in these analyses as recent studies in other cancers have demonstrated that loss of *PBRM1* is associated with increased T cell infiltration and response to ICI therapy [8, 9]. Indeed, Shrestha and colleagues report *PBRM1* as being among the top differentially expressed genes based on *BAP1* status [3]. Further studies are warranted to confirm the intriguing findings emerging from this integrated analysis of 19 PeM samples in larger, independent cohorts of PeM and to tease out the distinct effects of *BAP1* loss versus *PBRM1* loss on immune responses to PeM and PlM. In addition, the clinical validation of these findings will require correlation with data on ICI responses in relevant patient cohorts. Similarly, additional research to validate emerging immunotherapy targets, such as the protein “V-type immunoglobulin domain-containing suppressor of T-cell activation” (VISTA) [6], also known as V-set immunoregulatory receptor (VSIR), and identify predictive biomarkers should continue for all types of malignant mesothelioma**.**

## Abbreviations

ICI: Immune checkpoint inhibitor
PeM: Peritoneal mesothelioma
PlM: Pleural mesothelioma
TMB: Tumor mutational burden
